# Synthesis and Characterization of Redox-Active Charge-Transfer Complexes with 2,3,5,6-Tetracyanopyridine (TCNPy) for the Photogeneration of Pyridinium Radicals

**DOI:** 10.1002/chem.201201915

**Published:** 2012-12-11

**Authors:** Eva Wöß, Uwe Monkowius, Günther Knör

**Affiliations:** a Institute of Inorganic Chemistry, Johannes Kepler University Linz (JKU)Altenbergerstr. 69, A-4040 Linz, Austria

**Keywords:** charge transfer, donor–acceptor systems, photochemistry, proton transfer, radicals

## Abstract

The heteroaromatic polynitrile compound tetracyanopyridine (TCNPy) is introduced as a new electron acceptor for the formation of deeply colored charge-transfer complexes. In MeCN, TCNPy is characterized by a quasireversible one-electron-reduction process at −0.51 V (versus SCE). The tetracyanopyridine radical anion undergoes a secondary chemical reaction, which is assigned to a protonation step. TCNPy has been demonstrated to generate 1:1 complexes with various electron donors, including tetrathiafulvalene (TTF) and dihydroxybenzene derivatives, such as *p*-hydroquinone and catechol. Visible- or NIR-light-induced excitation of the intense charge-transfer bands of these compounds leads to a direct optical electron-transfer process for the formation of the corresponding radical-ion pairs. The presence of available electron donors that contain protic groups in close proximity to the TCNPy acceptor site opens up a new strategy for the photocontrolled generation of pyridinium radicals in a stepwise proton-coupled electron-transfer (PCET) sequence.

## Introduction

Unsaturated polynitrile compounds, such as tetracyanoethylene, 1,2,4,5-tetracyanobenzene, and related π-acceptor systems (TCNX), are well-established non-innocent ligands that result in redox-active compounds with a variety of interesting structural and physical characteristics, including magnetic behavior, conductivity, and long-wavelength optical absorption.^1^, ^2^ The much-less-studied title compound, 2,3,5,6-tetracyanopyridine (TCNPy), shares major structural similarities with these π-conjugated tetranitrile molecules. However, to the best of our knowledge, there are only two reports in the literature mentioning an unpublished synthesis and describing some preliminary characterization data on TCNPy.^3^, ^4^

Besides their important function as electron acceptors in charge-transfer (CT) systems,^4^ TCNX ligands display various modes of participation in metal-complex chemistry. They can coordinate in a π fashion or may also act as bridging ligands, thereby connecting up to four metal centers through σ bonds.^2^, ^5^, ^6^ In this context, TCNX ligands are either found to function as neutral π acceptors, as stable monoanionic radicals, or as dianions and, thus, can play an essential role as electron-transfer mediators in inorganic and organometallic chemistry.^5^, ^6^, ^7^, ^8^ In addition, the charge-transfer interactions of TCNX acceptor systems with various kinds of electron donors can lead to a wide variety of supramolecular binding motifs, including π—π stacking and the assembly of ionic layer structures.^2^, ^9^ These resulting materials frequently display very attractive functional properties, such as electrical conductivity, single-molecule magnetism, low-energy charge-transfer transitions, and non-linear optical activity.^2^, ^5^, ^10^, ^11^

**Scheme 1:**
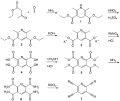
Synthesis of the heteroaromatic π-acceptor 2,3,5,6-tetracyanopyridine (7).

Whilst these various features of TCNX systems are interesting in their own right, the aza-substituted tetracyanobenzene derivative TCNPy (**7**), which carries a central pyridine moiety (Scheme 1), could also open new routes for applications in redox catalysis.

Recently, pyridinium derivatives and pyridinyl species have been discussed as potential redox mediators for the acceleration of net multielectron substrate conversions.^12^, ^13^ Protonation of the pyridine nitrogen atom and electrochemical one-electron reduction to form the corresponding pyridinium radical can lead to an efficient generation of hydrogen through disproportionation.^12^ Moreover, in the presence of carbon dioxide, pyridinium radicals—or coupling products thereof—have been postulated to participate in the stepwise electrocatalytic reduction of CO_2_ into MeOH under mild reaction conditions.^12^, ^13^ Plausible reaction mechanisms of this interesting proton-coupled redox process are currently under debate.

Herein, we decided to study the chemistry of 2,3,5,6-tetracyanopyridine (TCNPy; Scheme 1) with the aim of combining the excellent π-acceptor properties of aromatic TCNX-type polynitrile compounds with the intrinsic possibility of pyridinium-radical generation by exploiting suitable charge-transfer interactions in protic media.

## Results and Discussion

**Figure 1:**
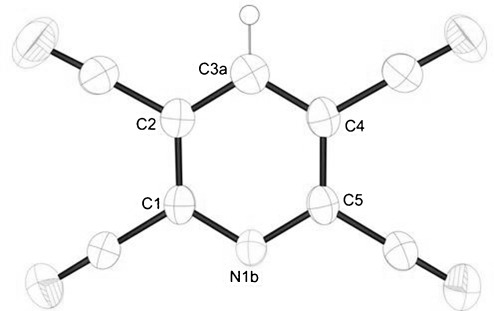
ORTEP of TCNPy (7); ellipsoids are set at 50 % probability. Only one orientation of the TCNPy moiety is shown.

The synthesis of 2,3,5,6-tetracyanopyridine (**7**) was achieved in 7 steps starting from ethyl acetoacetate, formaldehyde, and ammonia (Hantzsch pyridine synthesis^14^), as summarized in Scheme 1. After column chromatography on silica gel, analytically pure TCNPy was obtained in satisfactory yield as a colorless crystalline material, which was fully characterized by elemental analysis, FTIR and NMR spectroscopy. The molecular structure of compound **7** was determined by single-crystal X-ray diffraction (Figure 1).

**Figure 2:**
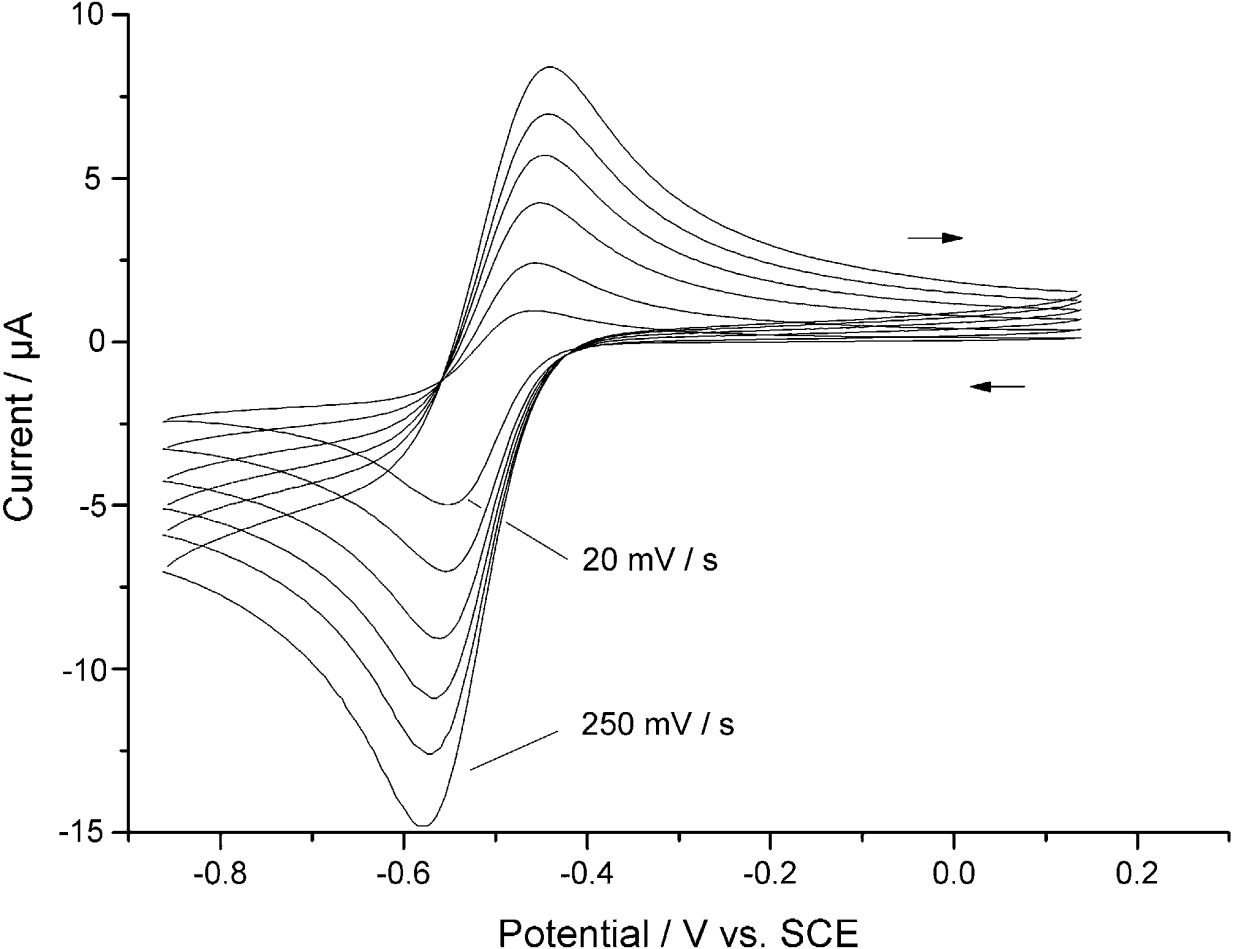
Cyclic voltammograms of 0.5 mm TCNPy in MeCN at room temperature with 0.1 m Bu_4_NPF_6_ supporting electrolyte and a Pt-disk working electrode; scan rates of 20, 50, 100, 150, 200, and 250 mV s^−1^ are shown.

The electron-accepting properties of TCNPy were studied by cyclic voltammetry (Figure 2). A quasireversible one-electron-reduction step occurs at a formal potential of *E*^o^*=*(*E*_pc_+*E*_pa_)/2=−0.51 V versus SCE (which corresponds to −0.27 V versus NHE or −4.3 eV versus the vacuum level).^15^ The separation between the cathodic and anodic peak potentials (Δ*E*_p_) increases with scan rate from 80 mV at 20 mV s^−1^ to 150 mV at 250 mV s^−1^. At the same time, a shift of the cathodic peak potentials (*E*_pc_) from −0.55 to −0.58 V is observed. The ratio between the cathodic peak current (*I*_pc_) and the square root of the scan rate (*υ*) initially decreases with increasing scan rate in a nonlinear fashion. Moreover, the ratio of anodic/cathodic peak current is less than unity and gradually increases to approximate *I*_pa_/*I*_pc_=1 at higher *υ* values. These diagnostic criteria indicate an electron-transfer process with a coupled homogeneous chemical-reaction step (EC mechanism)^16^, ^17^ that follows first-order kinetics (−30 mV shift of *E*_pc_ with Δlog *υ=*1 and *n*=1 at 298 K).

Furthermore, the set of cyclic voltammograms in Figure 2 is characterized by the presence of an isopoint at −0.56 V versus SCE, which allows us to estimate the electrochemical-transfer coefficient for the quasireversible process at low scan rates as *α*=0.55.^18^ Assuming a Stokes–Einstein radius of 5.5 Å for TCNPy and a kinematic viscosity of *η*=0.341 cP in MeCN at 298 K,^19^ the diffusion coefficient of compound **7** can be estimated as *D*=1.2×10^−5^ cm^2^ s^−1^. A standard heterogeneous electron-transfer rate constant of *k*^o^=5×10^−3^ cm s^−1^ was obtained according to the method of Nicholson,^20^ which indicated a substantial contribution of the inner-sphere-reorganization energy (*λ*_i_) to the electron-transfer process, as is frequently the case in organic redox couples,^21^ including pyridine derivatives that contain strongly electron-withdrawing groups.^22^ Digital simulation of the CVs at different scan rates is consistent with these general assignments (see the Supporting Information).

**Figure 3:**
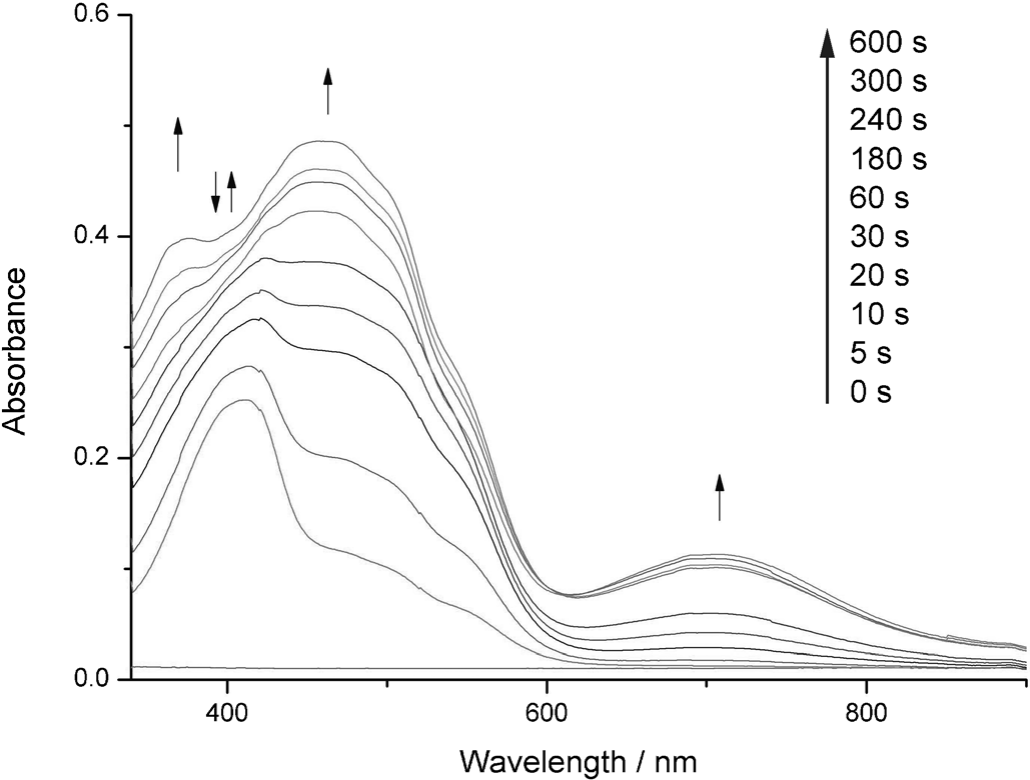
Changes in the UV/Vis absorption spectra of TCNPy in MeCN with 0.1 m Bu_4_NPF_6_ supporting electrolyte during the electrochemical reduction of TCNPy at −750 mV versus SCE.

During the course of the electrochemical reduction of TCNPy under thin-layer conditions in an OTTLE cell, the initially colorless solution of compound **7** rapidly turned orange. The corresponding spectroscopic variations that occurred at a controlled potential of −0.75 V versus SCE are shown in Figure 3.

The new absorption bands, which were observed within the first few seconds of the electrolysis, displayed a maximum at around 412 nm, which is within the typical range for a bathochromically shifted radical-anion spectrum of a pyridine derivative that contains electron-withdrawing groups (the radical anion of unsubstituted pyridine absorbs at 340 nm).^23^ At longer reaction times, the maximum at 412 nm disappears and new spectroscopic features arise, with peaks at 365, 470, and 707 nm (Figure 3). This behavior is consistent with a coupling of the radical-anion-generation step to a chemical secondary process (EC mechanism), as discussed above. The formation of dimerization products should be less favorable, according to the substitution pattern in compound **7**.^23^ This type of reaction would also be inconsistent with the observed first-order kinetics of the process. As an alternative mechanism, a pseudo-first-order protonation of the generated TCNPy^.−^ radical anion, which yields the corresponding pyridinium radical species, TCNPyH^.^, is tentatively suggested to account for the red-shifted spectrum of the secondary product, with a new peak maximum at 470 nm.

**Figure 4:**
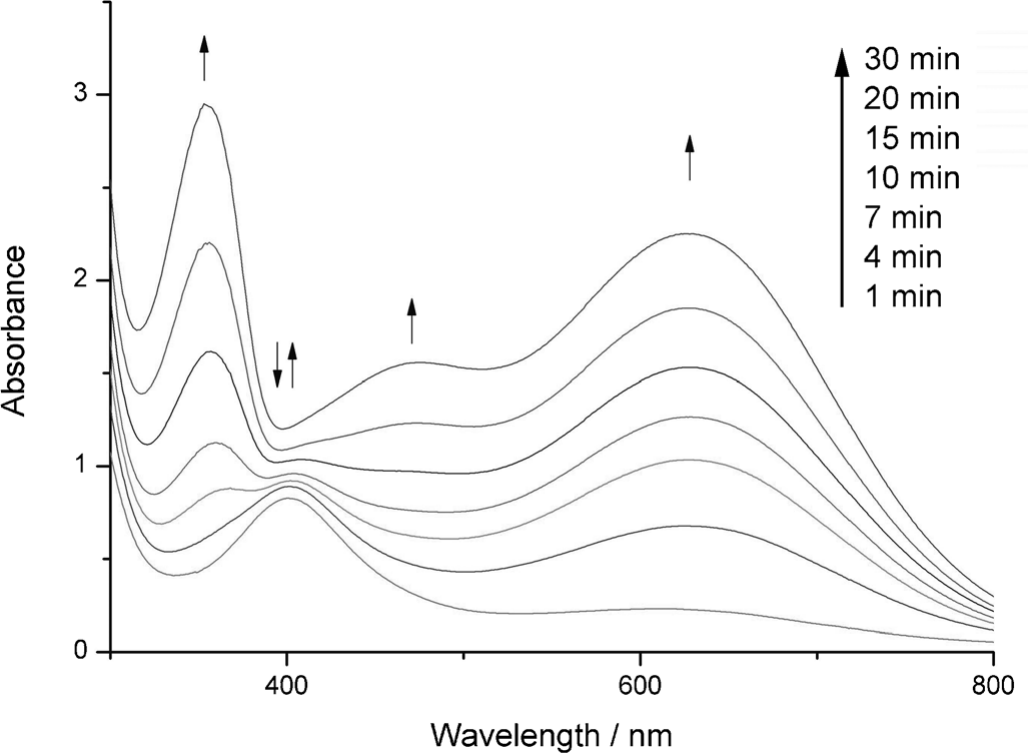
Chemical reduction of TCNPy with an excess of sodium amminepentacyanoferrate(II) in deionized water.

The reduction of compound **7** to generate TCNPy^.−^ could also be chemically induced in aqueous solution (Figure 4). For this purpose, we applied an excess of a pentacyanoferrate(II)–ammine complex, which acted as a one-electron donor (*E*°*=*0.34 V versus NHE, *λ*_max_=362 nm).^24^ Quite similar initial spectroscopic variations to those observed during the electrochemical reduction of TCNPy (Figure 3) were also obtained for this chemically induced redox process (Figure 4).

At room temperature, the reaction mixture gradually changed color from yellowish orange to green and finally dark blue. Whereas the initial signature of the pyridine radical anion at around 410 nm completely disappeared during the course of the reaction, other absorption maxima grew at 360, 468, and 626 nm (Figure 4). Similar to the electrochemical reaction in MeCN, this transition from 410 to 468 nm might indicate a protonation of the TCNPy^.−^ radical anion. However, owing to the presence of an excess of ferrous metal salt in solution, other possibilities, such as the involvement of metal-to-ligand charge-transfer (MLCT) transitions, also have to be considered, because further strong absorption bands were clearly overlapping with the spectroscopic features occuring in the corresponding electrochemical reduction of TCNPy. After a while, colloidal particles could be observed in the reaction mixture and, finally, a blue precipitate was obtained. This behavior strongly indicates the gradual formation of insoluble mixed-valence iron species, such as Prussian blue derivatives^25^ or related solid materials that involve the TCNX ligand,^26^ which should give rise to characteristic broad intervalence-charge-transfer (IVCT) or metal-to-metal charge-transfer (MMCT) absorption bands in the region 600–800 nm. However, any attempts to isolate and further characterize these presumably formed mixed-valence compounds, were beyond the scope of this work.

**Figure 5:**
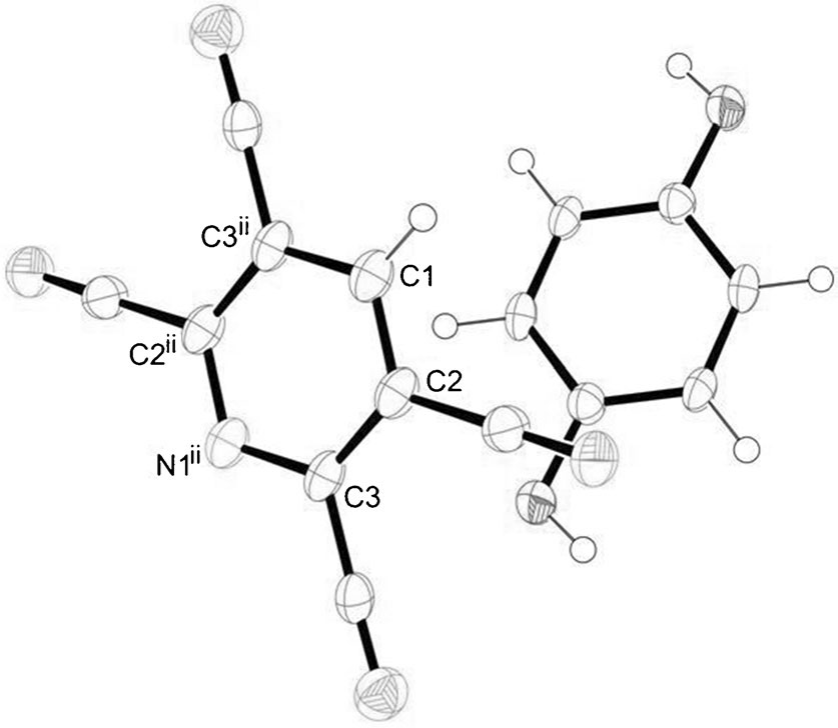
Molecular structure of a TCNPy—hydroquinone complex (8); symmetry code (ii): −*x*, −*y*+1, −*z*. Only one orientation of the distorted TCNPy moiety is shown (ORTEP, 50 % probability).

We also decided to study the electron-donor–acceptor interactions of compound **7** with a series of organic reductants, such as dihydroxybenzene derivatives and other diamagnetic compounds with low ionization energies. A red hydroquinone adduct (**8**) and an orange catechol complex (**9**) were readily obtained as solid crystalline materials. The molecular structure of the corresponding TCNPy complex with 1,4-dihydroxybenzene (**8**) is shown in Figure 5. Details on selected bond lengths and crystallographic data of compounds **7** and **8** are reported in Table 1 and Table 2.

**1:** Selected bond lengths in compounds 7 and 8.

Bond length [Å]	**7**	**8**
N1b—C1/N1^ii^—C3	1.365(3)	1.370(3)
C1—C2	1.393(3)	1.365(3)
C2—C3a/C2—C3	1.349(3)	1.387 (4)
C3a—C4/C1—C3^ii^	1.348(3)	1.370(3)
C4—C5/C2^ii^—C3^ii^	1.403(3)	1.387 (4)
C5—N1b/C2^ii^—N1^ii^	1.363(3)	1.365(3)
perimeter of the pyridine core[a]	8.221	8.244

[a] Sum of the bond lengths in the pyridine ring.

**Table 2:** Crystal data, data collection, and structure refinement for compounds 7 and 8.

	**7**	**8**
formula	C_9_HN_5_	C_15_H_7_O_2_N_5_
*M*_w_ [g mol^−1^]	179.15	289.26
crystal size [mm]	0.23×0.31×0.48	0.09×0.25×0.35
crystal system	orthorhombic	monoclinic
space group	*Pbca*	*P*2_1_/*n*
*a* [Å]	10.145(1)	6.646(2)
*b* [Å]	12.253(2)	6.563(1)
*c* [Å]	13.952(2)	15.190(4)
*β* [°]	–	101.63(1)
*V* [Å^3^]	1734.3(4)	649.0(3)
*ρ*_calcd_ [mg cm^−3^]	1.372	1.480
*Z*	8	2
*μ* [mm^−1^]	0.093	0.105
*T* [K]	205	200
*θ* range [°]	2.9-25.1	2.7-25.0
*λ* [Å]	0.71073	0.71073
total reflns	9941	3938
unique reflns	1539 (*R*_int_=0.064)	1142 (*R*_int_=0.068)
observed reflns [*I*>2*σ*(*I*)]	1137	880
refined parameters/restraints	128/0	101/0
absorption correction	multiscan
*T*_min_, *T*_max_	0.96, 0.98	0.95, 0.99
*σ*_fin_ (max/min) [e Å^−3^]	0.17/−0.17	0.18/−0.20
*R*_1_ [*I*≥2*σ*(*I*)]	0.049	0.048
*wR*_2_	0.110	0.118
CCDC no.	882755	882756

Upon the formation of a complex between compound **7** and hydroquinone, only minor structural changes in the TCNPy moiety were observed. Whereas the average CN bond length in the nitrile substituents remained essentially constant (1.14 Å), the perimeter of the planar aromatic core of TCNPy expanded slightly (<0.1 Å) when the adduct (**8**) was formed (Table 1). This lack of major bond-length alternations^3^ or other structural reorganization indicates a 1:1 charge-transfer interaction between the weakly coupled donor (D) and acceptor (A) systems in compound **8**, without a significant contribution of the antibonding π*-orbital character of the TCNPy subunit in the ground state.

**Figure 6:**
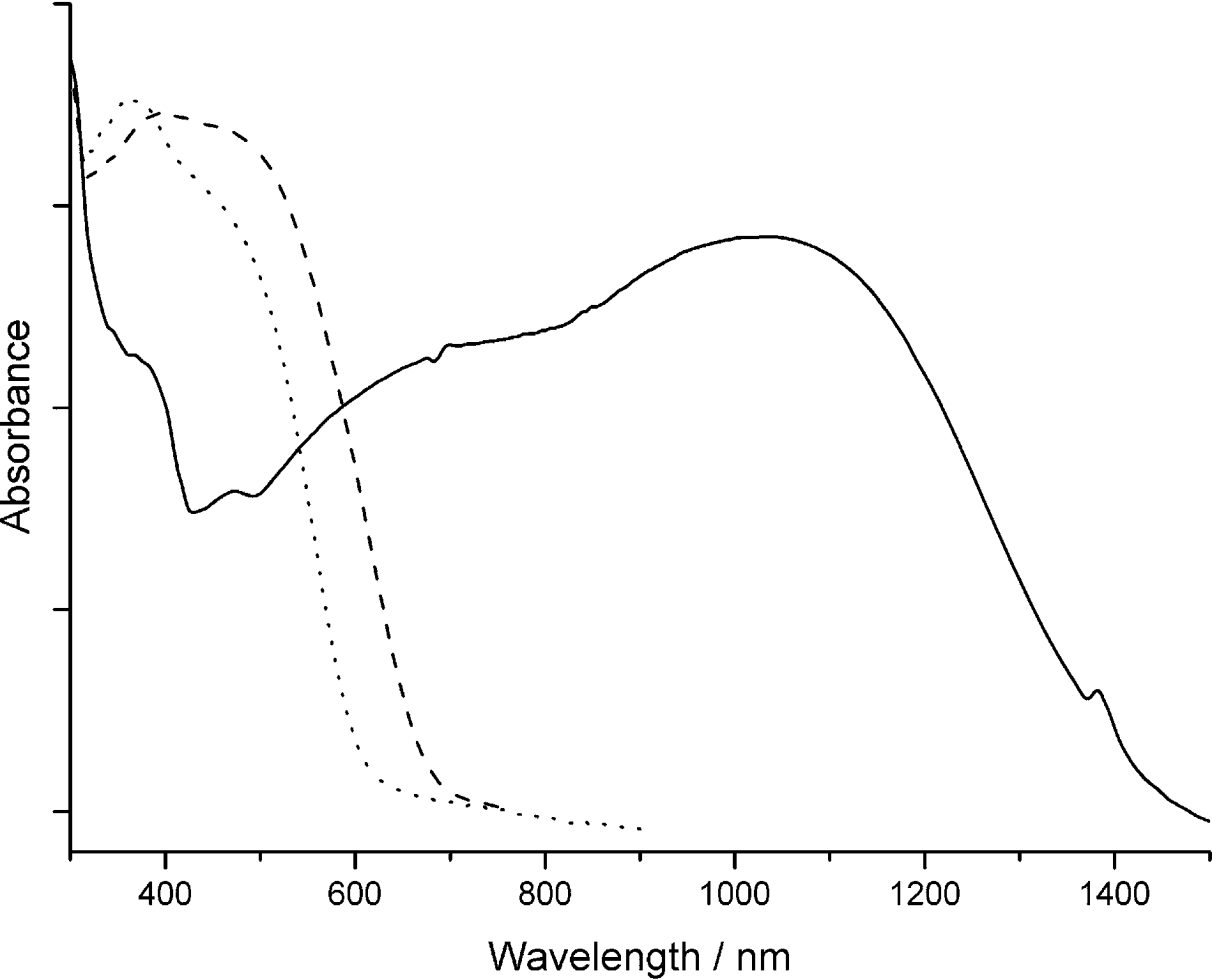
Diffuse reflectance spectra of compounds 8 (– – –), 9 (⋅⋅⋅), and 10 (—) at 298 K in a 5 wt. % mixture with BaSO_4_; the data are plotted as absorption spectra with baseline correction (BaSO_4_).

In Figure 6, the electronic spectra of charge-transfer complexes **8** and **9** are shown, together with the optical properties of the corresponding TCNPy adduct with tetrathiafulvalene (TTF) as an electron donor (**10**), which was obtained as a solid blue material. The chromophoric charge-transfer (CT) transitions of the TCNPy—dihydroxybenzene derivatives (**8** and **9**) appear as unresolved bands with absorption maxima in the range 500–600 nm. The deep-blue-colored TCNPy—TTF system (**10**) is characterized by a very broad absorption band with a maximum in the NIR spectroscopic region (*λ*_CT_=1050 nm). Interestingly, there is no indication of the presence of a TTF^.+^ radical cation in the electronic ground state of compound **10**, which could otherwise be easily identified by a characteristic spectroscopic pattern at 450 nm and 580 nm.^27^, ^28^ Quite similar spectroscopic features have been reported before at low temperatures in solution for other TTF-containing donor–acceptor, [D—A], systems.^28^ From the spectroscopic signatures shown in Figure 6, we conclude that, in compound **10**, a weakly coupled donor–acceptor charge-transfer state remains the dominant species, even at room temperature. TTF is a rather strong electron donor (*E*°*=*0.37 V versus SCE)^27^ and the driving force for an electron-transfer process to form the TCNPy^.−^ radical anion can be readily estimated (from our electrochemical data) as an endergonic process with Δ*G*_ET_=*F*(*E*°_ox_*−E*°_red_)=84 kJ m^−1^.

In contrast to some TTF-based systems with almost isergonic Δ*G*_ET_ values, which have been studied in detail by Kochi and co-workers,^28^, ^29^ in the electronic ground state of complex **10** there is no experimental evidence for more-strongly coupled [D^*δ*+^—A^δ−^] charge-transfer interactions with a significant degree of electronic delocalization, or even for the formation of a fully charge-separated [D^.+^,A^.+^] complex, which consists of a radical ion pair. At room temperature, the same situation should be true for the corresponding TCNPy—dihydroxybenzene systems, such as compound **8**, where the presence of a weakly coupled [D—A] charge-transfer (CT) state rather than an electron-transfer (ET) state with radical-ion-pair character has been assigned, owing to a lack of significant bond-length variations in the electron acceptor (see above; Table 1).

**Scheme 2:**
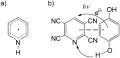
Pyridinium radical core of the reduced and protonated [TCNPyH]^.^ species. b) Representation of the weakly coupled [DH—A] charge-transfer ground state of compound 8, which illustrates the possibility of pyridinium-radical formation by coupling a direct optical electron-transfer process and a proton-transfer step.

However, according to the Mulliken theory, the direct optical excitation of the charge-transfer bands of such weakly coupled donor–acceptor pairs should immediately generate their corresponding electron-transfer states, [D^.+^,A^.−^]*.^29^ This non-adiabatic process for the formation of the donor radical cation and the acceptor radical anion can be exploited for the formation of permanent secondary redox products, when further chemical steps are successfully coupled within the lifetime of the charge-separated species. By using the heteroaromatic polynitrile system TCNPy as the electron acceptor (A), in combination with suitable proton-carrying electron donors (DH), such as dihydroxybenzene derivatives, this approach could offer interesting new routes for the visible- or NIR-light-controlled generation of pyridinium radical species, as outlined in Scheme 2.

The uptake of a proton to generate a neutral radical (AH^.^) is a typical stabilizing step of short-lived radical anions (A^.−^) in solution, which has already been discussed for the electrochemically generated TCNPy^.−^ intermediate, thereby producing a pyridinium radical species (see above). On the other hand, deprotonation is a very common decay mechanism of aromatic cation radicals that contain protic substituents. Recently, the primary proton-transfer reactions of *o*- and *p*-dihydroxybenzene radical cations of relevance for compounds **8** and **9** have been characterized for the first time in a detailed pulse-radiolysis study.^30^ It was found that these metastable donor radical cations (DH^.+^) decayed into their corresponding neutral semiquinone radicals (D^.^) with lifetimes of several hundreds of nanoseconds in nonpolar media. However, when polar proton-accepting compounds were present, the deprotonation step turned into a much more rapid process, thereby reaching the diffusion-controlled limit.

As shown in Scheme 2 b and Equation (1), the pre-requisites for such a sequential proton-coupled electron-transfer (PCET) cascade are fulfilled in donor–acceptor systems such as compound **8**, which is a charge-transfer complex that combines a proton-carrying donor (DH) and an electron acceptor (A) that can be protonated.

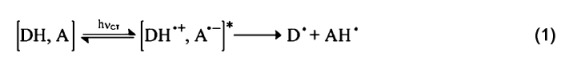


## Conclusion

Herein, we have reported an investigation into the synthesis, structural features, and extended characterization of the aromatic polynitrile compound 2,3,5,6-tetracyanopyridine (TCNPy). The electro- and spectroelectrochemical behavior, chemical reactivity, and electron-acceptor properties of TCNPy were studied in some detail. In MeCN, TCNPy is reduced into its corresponding radical anion at −0.51 V versus SCE (−4.3 eV versus the vacuum level), which most probably undergoes subsequent protonation into the neutral TCNPyH^.^ pyridinium radical. The interactions of TCNPy with metal complexes and the formation of organic donor–acceptor systems have also been investigated. Depending on the strength of the corresponding electron donor, several of these compounds show intense chromophoric absorption bands in the visible- and NIR spectroscopic regions; according to bond-length variation and diagnostic spectroscopic features, this result is ascribed to the presence of weakly coupled donor–acceptor charge-transfer states.

Photoexcitation of the donor–acceptor complexes by the direct irradiation of the charge-transfer absorption bands corresponds to an optical electron-transfer process; this process can be applied to generate the charge-separated excited state [D^.+^,A^.−^]*, which contains the TCNPy^.−^ radical-anion fragment. It is expected that, upon a subsequent protonation step, similar to the reactivity that was postulated under cyclovoltammetry conditions (EC mechanism), the corresponding TCNPy-derived pyridinium radical can be generated in a proton-coupled electron-transfer process, especially when pre-organized proton donors are present in close proximity to the acceptor site. Further study of the TCNPy derivatives and their charge-transfer complexes in the context of pyridinium-radical-dependent applications in electro-, photoelectro-, and photocatalysis of multielectron-reduction processes,^12^, ^13^, ^31^ such as dihydrogen generation or carbon-dioxide recycling, are currently underway.

## Experimental Section

**General methods**: All commercially available chemicals and solvents were reagent-grade quality and used as received without any further purification. NMR spectroscopy was performed on a Bruker Digital Avance NMR spectrometer DPX200 (^1^H: 200.1 MHz, ^13^C: 50.3 MHz; *T*=303 K). Chemical shifts are given in parts per million (ppm) on the delta (*δ*) scale. ^1^H and ^13^C NMR shifts are reported relative to Si(CH_3_)_4_ and were referred internally to the residual signal of the deuterated solvent. Coupling constants are given in Hertz (Hz). IR spectroscopy was performed on a Shimadzu IRAffinity-1 FTIR spectrophotometer that was equipped with a Specac Golden Gate^TM^ single-reflection diamond ATR accessory. Elemental analysis was performed at the Institut für Technologie Organischer Stoffe (Universität Linz).

**Electrochemical measurements**: Cyclic voltammetry (CV) experiments were performed on an Eco Autolab potentiostat at scan rates in the range 20–250 mV s^−1^. A standard three-electrode arrangement was used with a Pt-disk working electrode (BAS, surface area (*A*): 0.020 cm^2^), a Pt-wire counter electrode, and a Ag-wire reference electrode in MeCN with 0.1 m Bu_4_NPF_6_ supporting electrolyte. Ferrocene was used as an internal standard for potential referencing and the potentials were subsequently referenced versus the SCE. Digital simulations of the CVs were carried out with the BAS DigiSim® 3.03 software package. Spectroelectrochemistry was performed in an optically transparent thin-layer electrode (OTTLE cell)^32^ with a Pt working electrode, a Pt-wire counter electrode, and a Ag reference electrode. The corresponding absorption spectra were recorded on a Jasco V670 UV/Vis/NIR spectrophotometer.

**Electronic and diffuse reflectance spectroscopy**: Electronic absorption spectra were recorded in a 1 cm quartz cell on a Varian Cary 300 Bio UV/Vis spectrophotometer. Diffuse reflectance spectra were recorded on a Jasco V670 UV/Vis/NIR spectrometer that was equipped with an ISN-723 integrating-sphere attachment (inside diameter of the integrating sphere: 60 mm). BaSO_4_ was used for baseline correction and the samples were prepared as a 5 wt. % mixture of the compound in BaSO_4_. The resulting data were converted into absorption spectra by using Spectra Manager®.

**Crystal structures**: Single-crystal structure analysis was carried out on a Bruker Smart X2S diffractometer that was operating with Mo_Kα_ radiation (*λ*=0.71073 Å). Further crystallographic and refinement data can be found in Table 2. The structures were solved by using direct methods (SHELXS-97^33^) and refined by full-matrix least-squares on *F*^2^ (SHELXL-97^34^). The H atoms were calculated geometrically and a riding model was applied during the refinement process. In both compounds, the TCNPy moieties were found to be disordered and the pyridine N atom and the *para*-C atoms were occupied by both N and C atoms. Therefore, the structures were refined with an equal occupation of N and C atoms at each of the two positions by using the EXYZ and EADP commands.

CCDC-882755 http://www.ccdc.cam.ac.uk/cgi-bin/catreq.cgi(**7**) and CCDC-882756 http://www.ccdc.cam.ac.uk/cgi-bin/catreq.cgi(**8**) contain the supplementary crystallographic data for this paper. These data can be obtained free of charge from The Cambridge Crystallographic Data Centre via http://www.ccdc.cam.ac.uk/data_request/cif.

**Synthesis**: TCNPy was prepared in analogy to similar reaction steps reported in the literature.^35^, ^36^, ^37^

**Diethyl-1,2-dihydro-2,6-dimethylpyridine-3,5-dicarboxylate (1)**: Ethyl acetoacetate (204 mL, 1.6 mol), aqueous formaldehyde (37 %, 60 mL, 0.8 mol), and diethyl amine (1.2 mL, 0.01 mol) were stirred for 6 h at 0 °C and for 40 h at RT. The organic layer was separated and dried and EtOH (200 mL) was added. At 0 °C, NH_3_ gas was passed through this solution until saturation occurred and the mixture was stirred for 40 h at RT. Compound **1** (yellow precipitate) was filtered, washed with EtOH, and dried. Yield: 121.5 g (63 %); ^1^H NMR (200 MHz, DMSO, 20 °C, TMS): *δ*=8.25 (s, 1 H; NH), 4.06 (q, ^3^*J*(H,H)=7 Hz, 4 H; Et), 3.11 (s, 2 H), 2.11 (s, 6 H; Me), 1.19 ppm (t, ^3^*J*(H,H)=7 Hz, 6 H; Et); IR (ATR): 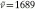 (C–O), 1209 cm^−1^ (C—O).

**Diethyl-2,6-dimethylpyridine-3,5-dicarboxylate (2)**: A cooled solution (5 °C) of water (150 mL), concd HNO_3_ (27 mL), and concd H_2_SO_4_ (22.5 mL) was added to compound **1** (106.2 g, 0.42 mol) and the mixture was heated at 90 °C for 20 min. After cooling, water (400 mL) was added and the resulting solution was neutralized with aqueous NH_3_. Compound **2** (gray precipitate) was filtered, washed with water, and dried. Yield: 98 g (93 %); ^1^H NMR (200 MHz, DMSO, 20 °C, TMS): *δ*=8.51 (s, 1 H; Ar), 4.33 (q, ^3^*J*(H,H)=7 Hz, 4 H; Et), 2.76 (s, 6 H; Me), 1.33 ppm (t, ^3^*J*(H,H)=7 Hz, 6 H; Et); IR (ATR): 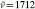 (C–O), 1220 cm^−1^ (C—O).

**Dipotassium-2,6-dimethylpyridine-3,5-dicarboxylate (3)**: Compound **2** (98 g, 0.39 mol) was dissolved in EtOH (300 mL) and a solution of KOH (52.8 g, 1.03 mol) in EtOH (300 mL) was added dropwise, followed by heating at reflux for 1 h. After cooling to RT, compound **3** (yellow precipitate) was filtered, washed with EtOH, and dried. Yield: 73.58 g (69 %); ^1^H NMR (200 MHz, DMSO, 20 °C, TMS): *δ*=7.69 (s, 1 H; Ar), 2.52 ppm (s, 6 H; Me); IR (ATR): 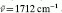 (C–O).

**Pyridine-2,3,5,6-tetracarboxylic acid (4)**: Compound **3** (15 g, 55 mmol) was heated at reflux with KMnO_4_ (71.4 g, 452 mmol) in water (500 mL) for 90 min. Excess KMnO_4_ was treated with EtOH and the hot mixture was filtered. Concd HCl was added to the filtrate until it reached pH 1, followed by evaporation of the solution to afford a mixture of compound **4** and KCl. ^1^H NMR (200 MHz, D_2_O, 20 °C, TMS): *δ*=8.84 ppm (s, 1 H; Ar); IR (ATR): 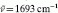 (C–O).

**Tetramethylpyridine-2,3,5,6-tetracarboxylate (5)**: Concd H_2_SO_4_ (15 mL) was added to a cooled suspension (0 °C) of compound **4** (14 g, 55 mmol) in MeOH (160 mL), followed by heating at reflux for 12 h. After cooling to RT, the solvent was evaporated and a saturated aqueous solution of NaHCO_3_ was added until it reached pH 8. Compound **5** was extracted from the mixture with EtOAc. Yield: 3.26 g (19 %); ^1^H NMR (200 MHz, DMSO, 20 °C, TMS): *δ*=8.75 (s, 1 H; Ar), 3.92 ppm (s, 12 H; Me); IR (ATR): 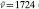 (C–O), 1240 cm^−1^ (C—O).

**Pyridine-2,3,5,6-tetracarboxamid (6)**: Compound **5** (3 g, 10 mmol) and NH_4_Cl (0.18 g, 3.5 mol) were dissolved in aqueous NH_3_ (28 mL) and stirred for 12 h at RT. Compound **6** was removed by filtration and dried. Yield: 2.29 g (94 %); ^1^H NMR (200 MHz, D_2_O/TFA 100:1, 20 °C, TMS): *δ*=7.50 ppm (s, 1 H; Ar); IR (ATR): 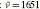 (C–O), 1581 cm^−1^ (C—N).

**2,3,5,6-Tetracyanopyridine (7)**: Compound **6** (2.2 g, 9.1 mmol) was dissolved in DMF (30 mL) at 0 °C under a N_2_ atmosphere. SOCl_2_ (3.0 mL, 42 mmol) was added dropwise, followed by stirring for 2 h at 0 °C and 12 h at RT. The reaction was quenched with water and crude TCNPy was extracted with EtOAc. After evaporation of the solvent, the reddish residue was purified by column chromatography on silica gel (CH_2_Cl_2_/Et_2_O, 1:1). TCNPy was afforded as a white powder. Yield: 1.17 g (72 %); ^1^H NMR (200 MHz, DMSO, 20 °C, TMS): *δ*=9.53 ppm (s, 1 H; Ar); ^13^C NMR (50 MHz, DMSO, 20 °C, TMS) *δ*=149.95 (*C*H), 141.43 (NC*C*N), 120.25 (NC*C*C), 116.81 (*C*N), 116.17 ppm (*C*N); IR (ATR): 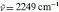 (C–N); elemental analysis calcd (%) for C_9_HN_5_: C 60.34, H 0.56, N 39.09; found: C 60.08, H 0.61, N 38.86.

**TCNPy—hydroquinone (8)**: Compound **7** (89.5 mg, 0.50 mmol) and hydroquinone (55 mg, 0.50 mmol) were stirred at RT in water (20 mL). Within 12 h, red crystals had formed and were removed by filtration. IR (ATR): 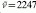, 1514, 1456, 1417, 1361, 1188, 1097, 1010, 920, 829, 756 cm^−1^; elemental analysis calcd (%) for C_15_H_7_N_5_O_2_: C 62.29, H 2.44, N 24.21; found: C 62.62, H 2.69, N 24.56.

**TCNPy—catechol (9)**: Compound **7** (89.5 mg, 0.50 mmol) and catechol (55 mg, 0.50 mmol) were stirred at RT in water (20 mL). Within 12 h, orange crystals had formed and were removed by filtration. IR (ATR): 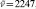, 1558, 1458, 1417, 1354, 1282, 1222, 1097, 1037, 931, 798 cm^−1^; elemental analysis calcd (%) for C_15_H_7_N_5_O_2_: C 62.29, H 2.44, N 24.21; found: C 62.47, H 2.71, N 24.19.

**TCNPy—tetrathiafulvalene (10)**: Compound **7** (89.5 mg, 0.5 mmol) and tetrathiafulvalene (102 mg, 0.5 mmol) were stirred at RT in MeCN (5 mL). Within 12 h, dark-blue crystals formed and were filtered. IR (ATR): 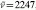, 1558, 1541, 1417, 1338, 1165, 1097, 925, 867, 792, 665 cm^−1^; elemental analysis calcd (%) for C_15_H_7_N_5_O_2_: C 46.98, H 1.31, N 18.26, S 33.45; found: C 46.51, H 1.33, N 17.98, S 33.18.

## Supporting Information

Detailed facts of importance to specialist readers are published as ”Supporting Information”. Such documents are peer-reviewed, but not copy-edited or typeset. They are made available as submitted by the authors.

## References

[1] Berger S, Hartmann H, Wanner M, Fiedler J, Kaim W (2001). Inorg. Chim. Acta.

[2] Kaim W, Moscherosch M (1994). Coord. Chem. Rev.

[3] Jones MT (1966). J. Am. Chem. Soc.

[4] Chen ECM, Wentworth WE (1975). J. Chem. Phys.

[5] Leirer M, Knör G, Vogler A (1999). Inorg. Chem. Commun.

[6] Maity AN, Schwederski B, Sarkar B, Záliš S, Fiedler J, Kar S, Lahiri GK, Duboc C, Grunert M, Gütlich P, Kaim W (2007). Inorg. Chem.

[7] Moscherosch M, Waldhör E, Binder H, Kaim W, Fiedler J (1995). Inorg. Chem.

[8] Olbrich-Deussner B, Kaim W, Gross-Lannert R (1989). Inorg. Chem.

[9] Wäckerlin C, Iacovita C, Chylarecka D, Fesser P, Jung TA, Ballav N (2011). Chem. Commun.

[10] Baumann F, Kaim W, Olabe JA, Parise AR, Jordanov J (1997). J. Chem. Soc., Dalton Trans.

[11] Glaser T (2011). Chem. Commun.

[12] Barton Cole E, Lakkaraju PS, Rampulla DM, Morris AJ, Abelev E, Bocarsly AB (2010). J. Am. Chem. Soc.

[13] Keith JA, Carter EA (2012). J. Am. Chem. Soc.

[14] Hantzsch A (1881). Chem. Ber.

[15] Bard AJ, Faulkner LR (2001). Electrochemical Methods: Fundamentals and Applications.

[16] Heinze J (1984). Angew. Chem.

[17] Nadjo L, Savéant JM (1973). J. Electroanal. Chem.

[18] Paul HJ, Leddy J (1995). Anal. Chem.

[19] Knör G (2000). Chem. Phys. Lett.

[20] Nicholson RS (1965). Anal. Chem.

[21] Grampp G, Rasmussen K (2002). Phys. Chem. Chem. Phys.

[22] Mohammad M, Aslam M (2011). J. Chem. Soc. Pak.

[23] Kalyanaraman V, Rao CNR, George MV (1971). J. Chem. Soc. B.

[24] Macartney DH (1988). Rev. Inorg. Chem.

[25] Itaya K, Uchida I, Neff VD (1986). Acc. Chem. Res.

[26] Diaz C, Arancibia A (2000). Polyhedron.

[27] Sun D, Rosokha SV, Kochi JK (2007). J. Phys. Chem. A J. Phys. Chem. B.

[28] Rosokha SV, Dibrov SM, Rosokha TY, Kochi JK (2006). Photochem. Photobiol. Sci.

[29] Rosokha SV, Kochi JK (2008). Acc. Chem. Res.

[30] Brede O, Kapoor S, Mukherjee T, Hermann R, Naumov S (2002). Phys. Chem. Chem. Phys.

[31] Knör G, Monkowius U (2011). Adv. Inorg. Chem.

[32] Krejčik M, Danđk M, Hartl F (1991). J. Electroanal. Chem.

[33] Sheldrick GM (1997). SHELXS-97, Program for the Solution of Crystal Structures.

[34] Sheldrick GM (1997). SHELXL-97, Program for Crystal Structure Refinement.

[35] Babu NJ, Nangia A (2006). Cryst. Growth Des.

[36] Yoshiizumi K, Yamamoto M, Miyasaka T, Ito Y, Kumihara H, Sawa M, Kiyoi T, Yamamoto T, Nakajima F, Hirayama R, Kondo H, Ishibushi E, Ohmoto H, Inoue Y, Yoshino K (2003). Bioorg. Med. Chem.

[37] Wöhrle D, Eskes M, Shigehara K, Yamada A (1993). Synthesis.

