# Supplementary material for: Synthesis and Characterization of Redox-Active Charge-Transfer Complexes with 2,3,5,6-Tetracyanopyridine (TCNPy) for the Photogeneration of Pyridinium Radicals
Source: Chemistry. 2012 Dec 11;19(4):1489–95. doi: 10.1002/chem.201201915 (PMC3564414; doi:10.1002/chem.201201915)

CHEMISTRY
A EUROPEAN JOURNAL

Supporting Information

© Copyright Wiley-VCH Verlag GmbH & Co. KGaA, 69451 Weinheim, 2013

Synthesis and Characterization of Redox-Active Charge-Transfer Complexes with 2,3,5,6-Tetracyanopyridine (TCNPy) for the Photogeneration of Pyridinium Radicals

Eva Wöß, Uwe Monkowius, and Günther Knör*[a]

chem_201201915_sm_miscellaneous_information.pdf

SUPPORTING INFORMATION

Synthesis and Characterization of Redox-Active Charge-Transfer Complexes with 2,3,5,6-Tetracyanopyridine (TCNPy) for the Photogeneration of Pyridinium Radicals

Eva Wöß, Uwe Monkowius, and Günther Knör*

1) Details on digital simulation of cyclic voltammograms:

Software used: DigiSim 3.03 (BAS Bioanalytical Systems)
Reference: A. W. Bott, *Current Seperations* **1999**, *18*, 9

EC-mechanism: A + e = B and B = C
CV-parameters: E(start) 0.1385 V ; E(rev) -0.862 V ; E(end) 0.1385 V
V / mV s^{-1} 0.02 ; 0.05 ; 0.10 ; 0.15 ; 0.20 ; 0.25
Cycles 1 ; T 298K ; area 0.02 cm^2 ; geometry: planar;
Chemical parameters: E° = -0.505 V ; α = 0.55 ; ks = 0.005 cm s^{-1} ; D = 1.2 x 10^{-5} cm^2 s^{-1}
K(eq) = 1 ; kf = 0.1

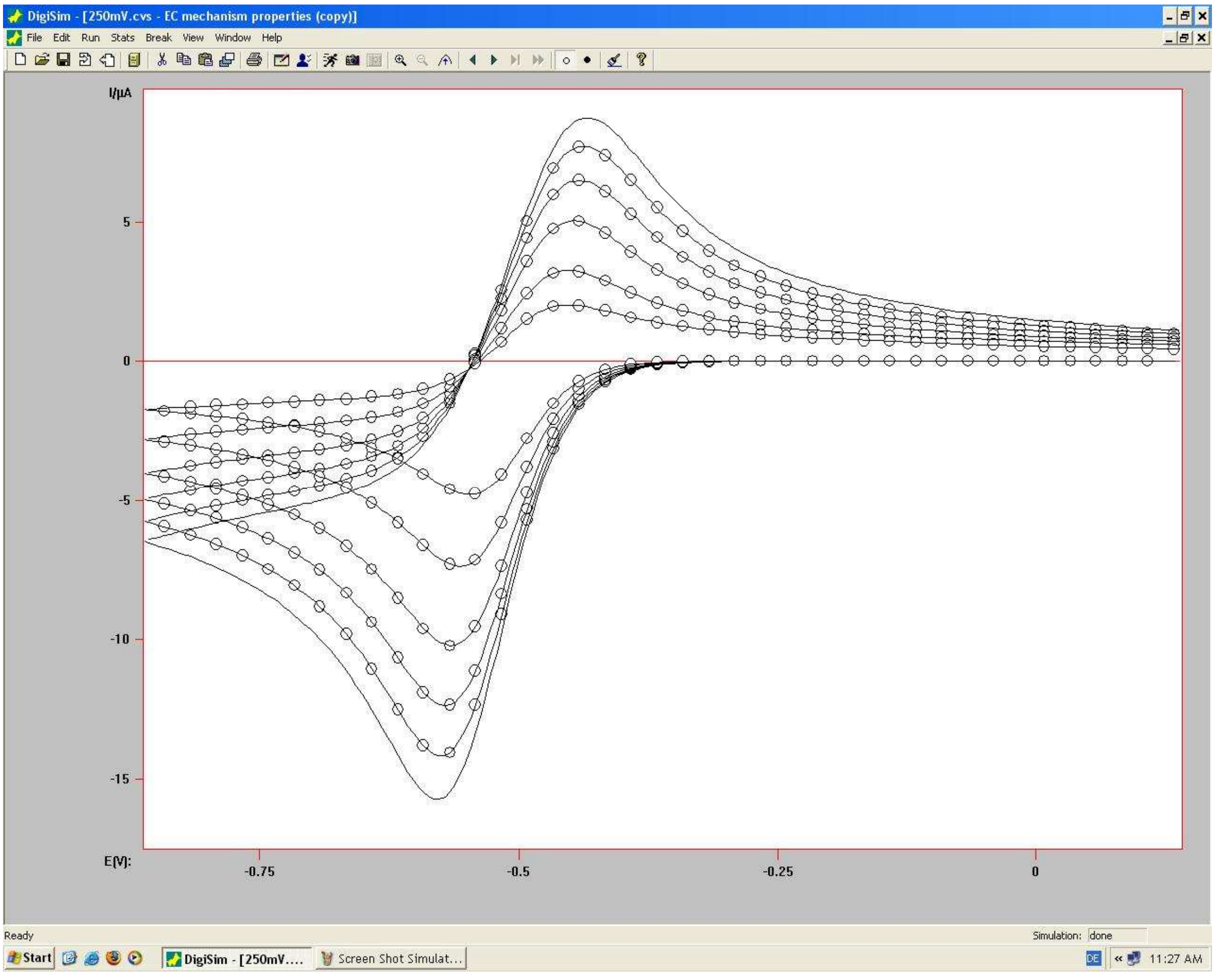

2) Details on X-ray structural determinations:

checkCIF/PLATON report

You have not supplied any structure factors. As a result the full set of tests cannot be run.

No syntax errors found. CIF dictionary Interpreting this report

Datablock: I

```
Bond precision:   C-C = 0.0030 A          Wavelength=0.71073

Cell:             a=10.1454(13)     b=12.2525(17)     c=13.952(2)
                  alpha=90          beta=90           gamma=90
Temperature:      205 K

                 Calculated                    Reported
Volume           1734.3(4)                     1734.3(4)
Space group      P b c a                       P b c a
Hall group       -P 2ac 2ab                    -P 2ac 2ab
Moiety formula   C9 H N5                       ?
Sum formula      C9 H N5                       C9 H N5
Mr               179.15                        179.15
Dx,g cm-3        1.372                         1.372
Z                8                             8
Mu (mm-1)        0.093                         0.093
F000             720.0                         720.0
F000'            720.21
h,k,lmax         12,14,16                      12,14,16
Nref             1542                          1539
Tmin,Tmax        0.966,0.979                   0.960,0.980
Tmin'            0.956

Correction method= MULTI-SCAN

Data completeness= 0.998          Theta(max)= 25.060

R(reflections)= 0.0491( 1137)     wR2(reflections)= 0.1102( 1539)

S = 1.175                   Npar= 128
```

The following ALERTS were generated. Each ALERT has the format
test-name_ALERT_alert-type_alert-level.
Click on the hyperlinks for more details of the test.

Alert level C

ABSTY02_ALERT_1_C An _exptl_absorpt_correction_type has been given without
a literature citation. This should be contained in the
_exptl_absorpt_process_details field.
Absorption correction given as multi-scan

```
PLAT048_ALERT_1_C MoietyFormula Not Given ........................          ?
PLAT366_ALERT_2_C Short? C(sp?)-C(sp?) Bond  C1     -   C2     ...       1.39 Ang.
PLAT420_ALERT_2_C D-H Without Acceptor      *N1B    -  *H3A    ...          ?
PLAT420_ALERT_2_C D-H Without Acceptor      *N1A    -  *H3B    ...          ?
```

Alert level G

```
PLAT005_ALERT_5_G No _iucr_refine_instructions_details in CIF ....          ?
PLAT007_ALERT_5_G Note: Number of Unrefined D-H Atoms ...........          2
PLAT194_ALERT_1_G Missing _cell_measurement_reflns_used datum ....          ?
PLAT195_ALERT_1_G Missing _cell_measurement_theta_max   datum ....          ?
PLAT196_ALERT_1_G Missing _cell_measurement_theta_min   datum ....          ?
PLAT301_ALERT_3_G Note: Main Residue  Disorder ..................         14 Perc.
```

---

0 **ALERT level A** = Most likely a serious problem - resolve or explain
0 **ALERT level B** = A potentially serious problem, consider carefully
5 **ALERT level C** = Check. Ensure it is not caused by an omission or oversight
6 **ALERT level G** = General information/check it is not something unexpected

5 ALERT type 1 CIF construction/syntax error, inconsistent or missing data
3 ALERT type 2 Indicator that the structure model may be wrong or deficient
1 ALERT type 3 Indicator that the structure quality may be low
0 ALERT type 4 Improvement, methodology, query or suggestion
2 ALERT type 5 Informative message, check

---

checkCIF publication errors

Alert level A

```
PUBL006_ALERT_1_A  _publ_requested_journal is missing
            e.g. 'Acta Crystallographica Section C'
```

---

1 **ALERT level A** = Data missing that is essential or data in wrong format
0 **ALERT level G** = General alerts. Data that may be required is missing

---

Publication of your CIF

You should attempt to resolve as many as possible of the alerts in all categories. Often the minor alerts point to easily fixed oversights, errors and omissions in your CIF or refinement strategy, so attention to these fine details can be worthwhile. In order to resolve some of the more serious problems it may be necessary to carry out additional measurements or structure refinements. However, the nature of your study may justify the reported deviations from journal submission requirements and the more serious of these should be commented upon in the discussion or experimental section of a paper or in the "special_details" fields of the CIF. *checkCIF* was carefully designed to identify outliers and unusual parameters, but every test has its limitations and alerts that are not important in a particular case may appear. Conversely, the absence of alerts does not guarantee there are no aspects of the results needing attention. It is up to the individual to critically assess their own results and, if necessary, seek expert advice.

If level A alerts remain, which you believe to be justified deviations, and you intend to submit this CIF for publication in Acta Crystallographica Section C or Section E, you should additionally insert an explanation in your CIF using the Validation Reply Form (VRF) below. Your explanation will be considered as part of the review process.

If you intend to submit to another section of Acta Crystallographica or Journal of Applied Crystallography or Journal of Synchrotron Radiation, you should make sure that at least a basic structural check is run on the final version of your CIF prior to submission.

```
# start Validation Reply Form
_vrf_PUBL006_GLOBAL
;
PROBLEM: _publ_requested_journal is missing
RESPONSE: ...
;
# end Validation Reply Form
```

If you wish to submit your CIF for publication in Acta Crystallographica Section C or E, you should upload your CIF via the web. If your CIF is to form part of a submission to another IUCr journal, you will be asked, either during electronic submission or by the Co-editor handling your paper, to upload your CIF via our web site.

PLATON version of 25/09/2012; check.def file version of 20/09/2012

Datablock I - ellipsoid plot

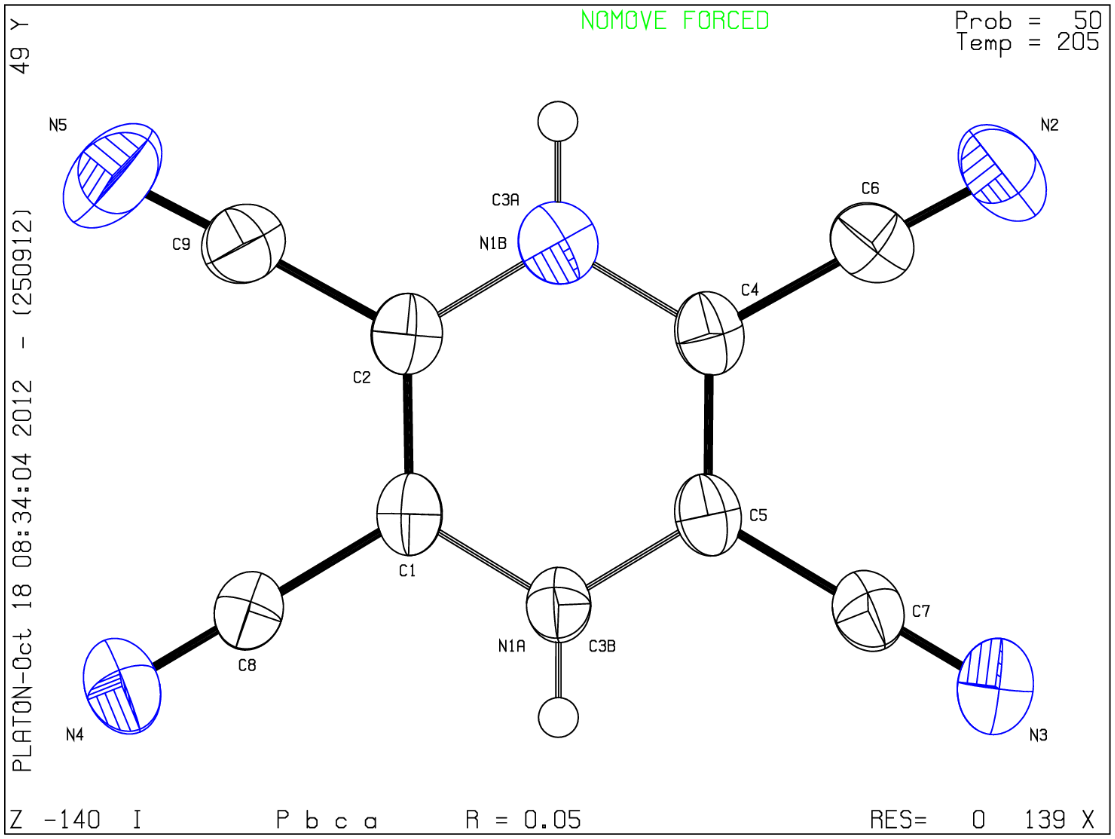

checkCIF/PLATON report

You have not supplied any structure factors. As a result the full set of tests cannot be run.

No syntax errors found. CIF dictionary Interpreting this report

Datablock: I

```
Bond precision:  C-C = 0.0033 A          Wavelength=0.71073

Cell:            a=6.6458(15)     b=6.5632(14)       c=15.190(4)
                 alpha=90         beta=101.630(8)    gamma=90
Temperature:     200 K

                Calculated                 Reported
Volume          649.0(3)                   649.0(3)
Space group     P 21/n                     P 1 21/n 1
Hall group      -P 2yn                     -P 2yn
Moiety formula  C9 H N5, C6 H6 O2          ?
Sum formula     C15 H7 N5 O2               C15 H7 N5 O2
Mr              289.26                     289.26
Dx,g cm-3       1.480                      1.480
Z               2                          2
Mu (mm-1)       0.105                      0.105
F000            296.0                      296.0
F000'           296.12
h,k,lmax        7,7,18                     7,7,18
Nref            1147                       1142
Tmin,Tmax       0.952,0.985                0.950,0.990
Tmin'           0.945

Correction method= MULTI-SCAN

Data completeness= 0.996          Theta(max)= 25.040

R(reflections)= 0.0480( 880)      wR2(reflections)= 0.1179( 1142)

S = 1.102                   Npar= 101
```

The following ALERTS were generated. Each ALERT has the format
test-name_ALERT_alert-type_alert-level.
Click on the hyperlinks for more details of the test.

Alert level C

ABSTY02_ALERT_1_C An _exptl_absorpt_correction_type has been given without a literature citation. This should be contained in the _exptl_absorpt_process_details field.
Absorption correction given as multi-scan

```
PLAT048_ALERT_1_C MoietyFormula Not Given ........................          ?
PLAT366_ALERT_2_C Short? C(sp?)-C(sp?) Bond  C2      -   C3      ...       1.39 Ang.
PLAT420_ALERT_2_C D-H Without Acceptor      *N1      -  *H1      ...          ?
```

Alert level G

```
PLAT005_ALERT_5_G No _iucr_refine_instructions_details in CIF ....          ?
PLAT007_ALERT_5_G Note: Number of Unrefined D-H Atoms ............          2
PLAT128_ALERT_4_G Alternate Setting of Space-group P21/c   .......      P21/n
PLAT194_ALERT_1_G Missing _cell_measurement_reflns_used datum ....          ?
PLAT195_ALERT_1_G Missing _cell_measurement_theta_max   datum ....          ?
PLAT196_ALERT_1_G Missing _cell_measurement_theta_min   datum ....          ?
PLAT301_ALERT_3_G Note: Main Residue  Disorder ...................          7 Perc.
PLAT371_ALERT_2_G Long   C(sp2)-C(sp1) Bond  C3      -   C5      ...       1.45 Ang.
PLAT779_ALERT_4_G Suspect or Irrelevant (Bond) Angle in CIF .... #          6
              N1   -C3   -C1      3.655   1.555   3.655             0.00 Deg.
```

---

0 **ALERT level A** = Most likely a serious problem - resolve or explain
0 **ALERT level B** = A potentially serious problem, consider carefully
4 **ALERT level C** = Check. Ensure it is not caused by an omission or oversight
9 **ALERT level G** = General information/check it is not something unexpected

5 ALERT type 1 CIF construction/syntax error, inconsistent or missing data
3 ALERT type 2 Indicator that the structure model may be wrong or deficient
1 ALERT type 3 Indicator that the structure quality may be low
2 ALERT type 4 Improvement, methodology, query or suggestion
2 ALERT type 5 Informative message, check

---

checkCIF publication errors

Alert level A

```
PUBL006_ALERT_1_A  _publ_requested_journal is missing
            e.g. 'Acta Crystallographica Section C'
```

---

1 **ALERT level A** = Data missing that is essential or data in wrong format
0 **ALERT level G** = General alerts. Data that may be required is missing

---

Publication of your CIF

You should attempt to resolve as many as possible of the alerts in all categories. Often the minor alerts point to easily fixed oversights, errors and omissions in your CIF or refinement strategy, so attention to these fine details can be worthwhile. In order to resolve some of the more serious problems it may be necessary to carry out additional measurements or structure refinements. However, the nature of your study may justify the reported deviations from journal submission requirements and the more serious of these should be commented upon in the discussion or experimental section of a paper or in the "special_details" fields of the CIF. *checkCIF* was carefully designed to identify outliers and unusual parameters, but every test has its limitations and alerts that are not important in a particular case may appear. Conversely, the absence of alerts does not guarantee there are no aspects of the results needing attention. It is up to the individual to critically assess their own results and, if necessary, seek expert advice.

If level A alerts remain, which you believe to be justified deviations, and you intend to submit this CIF for publication in Acta Crystallographica Section C or Section E, you should additionally insert an explanation in your CIF using the Validation Reply Form (VRF) below. Your explanation will be considered as part of the review process.

If you intend to submit to another section of Acta Crystallographica or Journal of Applied Crystallography or Journal of Synchrotron Radiation, you should make sure that at least a basic structural check is run on the final version of your CIF prior to submission.

```
# start Validation Reply Form
_vrf_PUBL006_GLOBAL
;
PROBLEM: _publ_requested_journal is missing
RESPONSE: ...
;
# end Validation Reply Form
```

If you wish to submit your CIF for publication in Acta Crystallographica Section C or E, you should upload your CIF via the web. If your CIF is to form part of a submission to another IUCr journal, you will be asked, either during electronic submission or by the Co-editor handling your paper, to upload your CIF via our web site.

PLATON version of 25/09/2012; check.def file version of 20/09/2012

Datablock I - ellipsoid plot

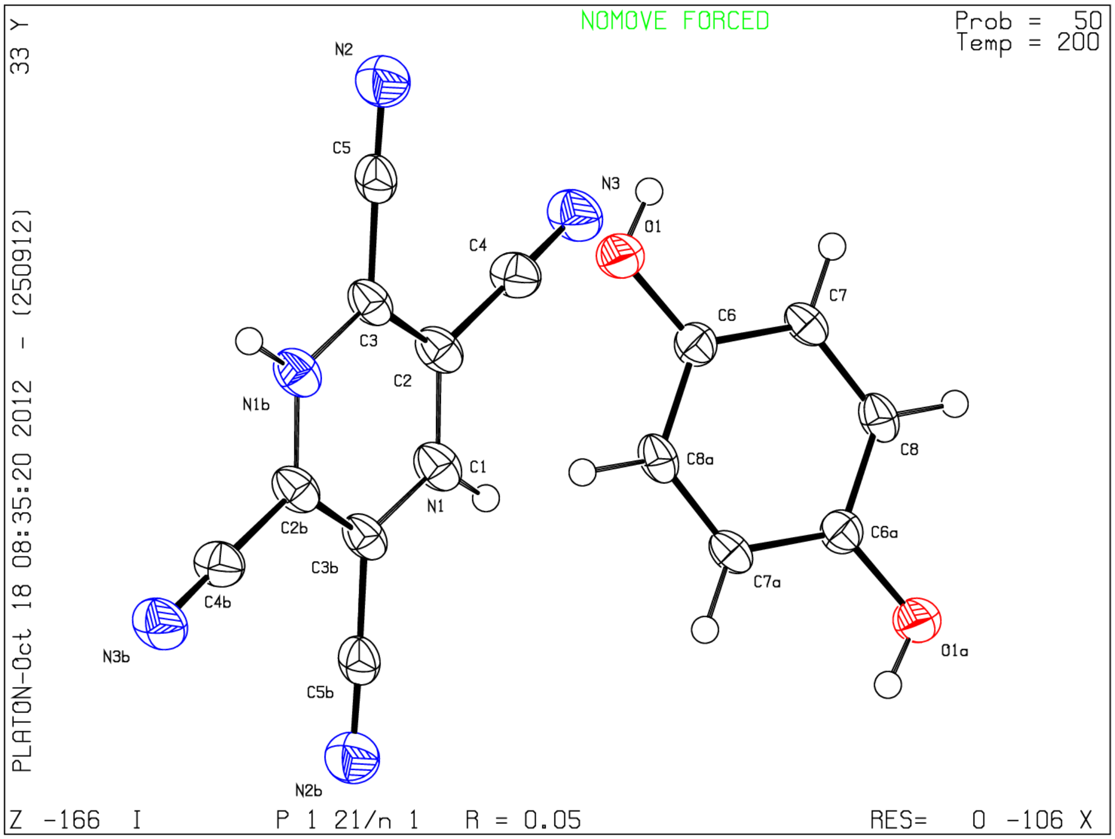

Supplement: Supplementary file 1 [file chem0019-1489-sd1.pdf]
